# Dr. Bird’s Report of the Cook County Hospital

**Published:** 1848-02

**Authors:** J. H. Bird

**Affiliations:** Attending Physician; Chicago


### ARTICLE V.

*Report of Cases treated in the Dispensary of Cook County Hospital, from September*, 1847, *to January*, 1848. By J. H. Bird, M. D., attending Physician.

| | | | |
|---|---|---|---|
| Amaurosis, | 1 | Hernia, | 2 |
| Amenorrhœa, | 4 | Infantile Remittent, | 6 |
| Asthma, | 2 | Leucorrhœa, | 2 |
| Bronchitis, | 13 | Menorrhagia, | 1 |
| Corneitis | 1 | Neuralgia, | 3 |
| Corjunctivitis | 3 | Pertussis, | 1 |
| Constipation, | 8 | Pleurisy, | 1 |
| Dentition, | 3 | Porrigo, | 6 |
| Dilatation of Bronchi, | 1 | Remittent Fever, | 23 |
| Diarrhœa, | 20 | Rheumatism, | 2 |
| Dropsy, | 1 | Sarsocele, | 1 |
| Dysentery, | 4 | Scabies, | 1 |
| Dysmenorrhœa, | 2 | Scrofula, | 4 |
| Gravel, | 1 | Syphilis primary, | 1 |
| Intermittent Fever. | | " Tertiary, | 1 |
| Tertian 65 | | Synovial Infl. | 1 |
| Quotidian, 45 | | Typhoid Fever, | 6 |
| | 110 | Worms, | 5 |
| | 174 | | 72 |
| | | | 174 |
| | | | 246 |

Intermittents were treated with quinine, salacine, cinchonine, singly, and in combination with the prussiate of iron. Quinine and Salacine combined, in the proportion of one part of the former to three of the latter, was administered with success in some cases. Strychnia was given in several cases, with less satisfactory results, than when administered for spring intermittents.

In cases accompanied with derangement of the stomach, salacine and cinchonine more effectually arrested the paroxysm than did quinine. Cinchonine was introduced and prescribed, during the last two months specified above. In doses similar to quinine it was quite as prompt in arresting the paroxysm. Being nearly tasteless. and less irritating than quinine, to the stomach, it is to be preferred, in many cases, where the latter, from its bitter taste and irritating qualities, would be rejected.

It has been given with quite as satisfactory results in the remittent fevers, that have been treated during the short period of its trial; also, in several cases of the diarrhœa, premonitory to an attack of fever. From the success that has been met with in its use, and from its being less expensive than quinine, (at two-thirds the cost,) it is a very desirable remedy in the treatment of miasmatic diseases.

The cases of remittent fever were generally free from complication; when occurring, they were then treated in the usual manner, according to the complication. The treatment, generally adopted, was a combination of quinine or cinchonine with opium, sometimes with the addition of ipecac, as the appearance of the tongue might indicate, following the operation of a mild cathartic.

The cases of typhoid fever were treated in a similar manner to remittants, by the use of quinine and opium, with the exception, that opium was administered in much larger doses and more frequently, together with a free use of stimulants.

Nitric acid drink was prescribed, both in this and remittent fever, where indicated by the character of evacuations, and, also, during convalescence.

In the cases of asthma, the severe dyspnœa was greatly relieved by the inhalation of sulphuric ether. The patients not reporting themselves, the results of its administration were not ascertained.

Chicago, Feb. 28, 1848.

